# Our Medical Literature

**Published:** 1882-01

**Authors:** John S. Billings

**Affiliations:** Surgeon U. S. Army


### SELECTIONS.

Often in difficult operations in very wet months the rubber dam will sometimes leak sufficient to endanger a filling. Copal ether varnish or a thin solution of gutta-percha stopping in chloroform, flowed around the neck of the tooth and edges of the dam, will effectually seal it.

*International Medical Congress*, 1881.

### OUR MEDICAL LITERATURE.

ADDRESS BY JOHN S. BILLINGS, M. D., SURGEON U. S. ARMY.

[CONTINUED.]

Now, while it is true that Professor Foster, in his address before the British Medical Association last year (which address is the clearest exposition of the aims of the physiology of the present day that I have seen), insists upon the fact that all distinctions between physiology and pathology are fictitious, and declares that attempts to divide them are like attempts to divide meterology into a science of good and a science of bad weather, his conclusion that the pathologist should be trained in methods of physiological investigation seems to me to be a part only of the truth. The tacit assumption is that all, or at least, the most important phenomena of human disease may be reproduced in the physiological laboratory. If this were only true, what a tremendous stride would have been taken towards making medicine a science! Unfortunately, it is not so. Many of the most interesting of these phenomena—the most interesting because as yet the most unexplainable—can only be observed in the sick man himself. Nor have the physiologists as yet made much use of that field which ought to be specially inviting to them, namely, comparative pathology; although the literature of the present time already indicates that a change has begun in this respect.

While it is true that to the graduate of thirty years ago much of the physiological literature of the present day is in an unknown tongue, it is also true that the physiologist of the present, who confines himself to laboratory work, will find himself distanced by the man who keeps his clinical and pathological studies and his experimental work well abreast.

The increase in both the amount and value of the literature of the several specialties in medicine is readily seen by a comparison of recent catalogues and bibliographies with those of twenty or thirty years ago, and this increase still continues at a greater rate than prevails in the more general branches. There are great differences of opinion as to the relative value of this increase and as to its future effect upon the profession, but there can be no doubt as to the fact. There must be specialties and specialists in medicine, and the results will be both good and evil; but the evils fall largely upon those specialists who have an insufficient general education---who attempt to construct their pyramid of knowledge with the small end as a foundation. It has been said by Dr. Hodgen that "in medicine a specialist should be a skilled physician and something more, but that he is often something else,---and something less." There is truth in this; truth which the young man will do well to consider with care before he begins to specialise his studies; but on the other hand it is also true that the great majority of men must limit their field of work very much and very clearly if they hope to achieve success. The tool must have an edge if it is to cut. It is by the labor of specialists that many of the new channels for thought and research have been opened, and if the flood has sometimes seemed to spread too far, and to lose itself in shallow and sandy places, it has nevertheless tended to fertilize them in the end.

The specialists are not only making the principal advances in science, but they are furnishing both strong incentives and valuable assistance towards the collection and preservation of medical literature and the formation of large public libraries.

Burton declares that a great library cannot be improvised, not even if one had the national debt to do it with; thinks that 20,000 volumes is about the limit of what a miscellaneous collection can bring together, and refers especially to the difficulty in creating large public libraries in America. My experience would show that these statements do not apply to medical books. Of these the folios and quartos of three and four hundred years ago seem to have had great capacity for resistance to ordinary destructive forces. Perhaps much of this is due to the fact that they are not usually injured by too much handling or perusal. True, they are gradually becoming rarer, but at the same time by means of properly organized libraries they are becoming more accessible to all who wish to really use them, and not merely to collect and hide them away. They drift about like the sea-weed, but the survivors are gradually finding secure aad permanent resting-places in the score of great collections of such literature which the world now possesses. At present the currents of trade are carrying them in relatively large numbers to the United States, where medical collectors and specialists are among the best customers of the antiquarian booksellers of Europe. I could name a dozen American physicians who have given to European agents almost unlimited orders for books relating to their several specialties, and upon their shelves may be found books of the fifteenth and sixteenth centuries, which may be properly marked as "rarissime."

Not that the rarest books are by any means the oldest. The collector who seeks to ornament his shelves with the "Rose of John of Gaddesden," or the "Lily of Bernard de Gordon," the first folios of Avicenna or Celsus, or almost any of the eight hundred medical *incunabulæ* described by Hain, will probably succeed in his quest quite as soon as the one who has set his heart on the first editions of Harvey or Jenner, the American tracts on inoculation for small-pox, or complete sets of many of the journals and transactions of the present century.

Whatever may be the chosen line of the book collector, he is the special helper of the public library, and this whether he intend it or not. In most cases his treasures pass through the auction room, and sooner or later the librarian, who can afford to wait, will secure them from further travel. Thanks to the labors of such collectors, I think it is safe to say---what certainly would not have been true twenty years ago---that if the entire medical literature of the world with the exception of that which is collected in the United States, were to be now destroyed, nearly all of it that is valuable could be reproduced without difficulty.

What is to be the result of this steadily increasing production of books? What will the libraries and catalogues and bibliographies of a thousand, or even of a hundred years hence, be like if we are thus to go on in the ratio of geometric progression which has governed the press for the last few decades? The mathematical formula which would express this, based on the data of the past century, gives an absurd and impossible conclusion, for it shows that if we go on as we have been going there is coming a time when our libraries will become large cities, and when it will require the services of every one in the world, not engaged in writing, to catalogue and care for the annual product. The truth is, however, that the ratio has changed, and that the rate of increase is becoming smaller. In Western Europe, which is now the great centre of literary production, it does not seem probable that the number of writers or readers will materially increase in the future; and it is in America, Russia, and Southern Asia that the greatest difference will be found between the present amount of annual literary product and that of a century hence.

The analogies between the mental and physical development of an individual, and of a nation or society, have been often set forth and commented on, but there is one point where the analogy fails as regards the products of mental activity---and that is, that as yet we have devised no process for getting rid of the exuviæ. Growth and development in the physical world imply the changes of death as well as of life---that with the increase of the living tissues there shall also be the excretion and destruction of dead, outgrown, and useless matters which have had their day and served their purpose. But---*litera scripta manet.* There is a vast amount of this effete and worthless material in the literature of medicine, and it is increasing rapidly. Our literature is in fact something like the inheritance of the Golden Dustman, but with this important difference, viz., that when the children raked a few shells or bits of bone from the dust-man's heap, and, after stringing them together and playing with them a little while, threw them back, they did not thereby add to the bulk of the pile; whereas our preparers of compilations and compendiums, big, and little, acknowledged or not, are continually increasing the collection, and for the most part with material which has been characterized as "superlatively middling, the quintessential extract of mediocrity." A large medical library is in itself discouraging to many inquirers, and I have become quite familiar with the peculiar expression of mingled surprise, awe, and despair which is apt to steal over the face of one not accustomed to such work when he first finds himself fairly in the presence of the mass of material which he wishes to examine for the purpose of completing his ideal bibliography of---let us say epilepsy, or excisions, or the functions of the liver.

Let such inquirers, as well as those who regret that they have no access to large libraries, and must therefore rely on the common text-books and current periodicals for bibliography, console themselves with the reflection that much the larger part of all our literature which has any practical value belongs to the present century, and, indeed, will be found in the publications of the last twenty years.

There are a few books written prior to 1800 which every well-educated medical man should---I will not say read, but---dip into, such as some of the works of Hippocrates and Galen, of Harvey and Hunter, of Morgagni and Sydenham; but this is to be done to learn their methods and style rather than their facts or theories, and by the great majority of physicians it can be done with much more profit in modern translations than in the originals. The really valuable part of the observations of these old masters has long ago become a part of the common stock, and the results are to be found in every text-book.

If, perchance, among the dusty folios there are stray golden grains yet ungleaned, remember that just in front are whole fields waiting the reaper. There is not, and has not been, any lack of men who have the taste and time to search the records of the past; and the man who has opportunities to make experiments or observations for himself, wastes his time, to a certain extent, if he tries to do bibliographical work so long as he can get it done for him. He wishes to know whether this problem has been attacked before, and with what result---whether there are accounts of any other cases like the one he has in hand. In ninety-nine instances out of a hundred, if the answer to these questions is not given in the current text-books or monographs, it is not worth prolonged search by the original investigator. Yet he should know how to make this search, if only to enable him to direct others, and it is for this reason that a little acquaintance with bibliographical methods of work ought to be obtained by the student.

When a physician has observed (or thinks he has observed) a fact, or has evolved from his inner consciousness a theory which he wishes to examine by the light of medical literature, he is often very much at a loss to know how to begin, even when he has a large library accessible for the purpose.

The information he desires may be in the volume next his hand, but how is he to know that? And even when the usual subject-catalogue is placed before him, he finds it very difficult to use it, especially when, as is often the case, he has by no means a well-defined idea as to what it is he wishes to look for. Upon the title-page of the " Washington City Directory " is printed the following aphorism :---" To find a name you must know how to spell it." This has a very extensive application in medical bibliography. To find accounts of cases similar to your own rare case you must know what your own case is.

To return to the subject-catalogue. If it is a classed catalogue---a *catalogue raisonne*---it will often seem to be a very blind guide to one who is not familiar with the classification and nomenclature adopted by the compiler. And certainly some of these classifications are very curious, reminding one of Heine's division of ideas into reasonable ideas, unreasonable ideas, and ideas covered with green leather. But if the inquirer has mastered the arrangement of the catalogue, it is two to one that it will not help him. It is a catalogue of the titles of books, but very often the title of a book gives very little information as to its contents, if, indeed, it is not actually misleading. Now, suppose the particular case he has in hand is one of a new-born infant having one leg much larger and longer than the other. He will find no book title relating to this. There may be a book in the library on diseases of the lymphatics which contains just what he wants; but unless he knows that his case is one affecting the lymphatics he will hardly get the clue. There may also be in the library twenty papers, in as many different volumes of journals and transactions, the titles of which show that they probably relate to similar cases, but the titles of such papers do not appear in the catalogue.

It should also be observed that subject-catalogues may easily be put to improper uses, or thought to give more information than they really do. They are not bibliographies, but mechanical aids in bibliographical work.

You will perhaps pardon me for taking as an illustration the index catalogue of the library of the Surgeon-General's Office, in Washington, as being one with which I am familiar, and which I can venture to comment on without risk of its being thought that I wish to depreciate its value. Taking any given subject in medicine, it is possible for a fairly-educated physician to obtain from this catalogue a large proportion of all the references which have any special value, and by so doing to save a vast amount of time and labor. On the other hand, he will find, when he comes to examine the books and articles referred to, that at least one-half of them are of no value so long as the other half are accessible, seeing that they are dilutions and dilatations, rehashes and summaries of the really original papers. If the seeker is in the library itself, this does not cause a great waste of time, as he can rapidly examine and lay aside those that do not serve his purpose. But if he is using this catalogue in another library---say, here in London---the case is different. It is highly improbable that he will find in any other collection all the books referred to; and then comes the annoyance of the doubt as to whether he may not be missing some very valuable paper. How is he to know whether or not Smith, in his pamphlet on the functions of the pneumogastric, has anticipated his own theory of its relations to enlarged tonsils? And in all such cases, *omne ignotum*

*est pro magnifico.* In a bibliography of the subject, prepared from the same material as the catalogue, he would either find no mention of Smith's paper, or, better still, a note that his paper is merely an abstract or compilation. The fact that he does not find Smith's book in the London Library, nor any allusion to it in the best works on the subject, ought to induce him to ignore it altogether.

In proportion to the energy of the young writer, and his determination to not only note everything that has been written about his subject, but to carry out the golden rule of verifying all his references, he is apt to be led off from his direct research into the many attractive by-paths of quaint and curious speculation which he will find branching off on every side; and this danger must be guarded against, or he will find that he is wasting his time and energy in turning over chaff which has long ago been pretty thoroughly threshed and winnowed.

It is, however, no part of my present purpose to set forth the methods and principles of bibliography---it is sufficient to point out their importance, and to call attention to the point that a knowledge of how and where to find the record of a fact is often of more practical use than a knowledge of the fact itself: just as we value an encyclopædia for occasional reference, and not for the purpose of reading through from cover to cover.

Instruction in the history and literature of medicine forms no part of the course of medical education in English and American schools, nor should I be disposed to recommend its introduction into the curriculum if it were to be based on French and German models, but it does seem possible to take a step in this direction which would be of great value, not only as a means of general culture, as teaching students how to think, but from a purely practical point of view, in teaching them how to use the implements of their profession to the best advantage---for books are properly compared to tools of which the index is the handle. Such instruction should be given in a library, just as chemistry should be taught in a laboratory. The way to learn history and bibliography is to make them; the best work of the instructor is to show his students how to make them.

In the absence of some instruction of this kind the student is liable to waste much time in bibliographical research. There has been much more done in this direction than many writers seem to suppose, and there are not many subjects in medicine which have not been treated from this point of view. Of course, all is not bibliography which pretends to be such. Very many of the exhaustive and exhausting list of references which are now so common in medical journal articles have been taken largely at second hand, and thereby originate or perpetuate errors. It is well to avoid false pride in this matter. To overlook a reference is by no means discreditable; but a wrong reference, or an unwitting reference to the same thing twice, gives a strong presumption of carelessness and second-hand work. Journal articles, however, and especially reports of cases, undergo strange transmogrifications sometimes, and I have watched this with interest in the case of a French or German paper, translated and condensed in the *London Record*, then appearing in abstract under the name of the translator in a leading journal, then translated again, with a few new circumstances, in a continental periodical, and finally, perhaps, reversed and appearing as an original contribution in the pages of the *Little Peddlington Medical Universe.*

In this connection it is well to remember that a mere accumulation of observations, no matter how great the number, does not constitute science, especially if these observations have been recorded under the influence of the same theories and in essentially similar conditions.

Science seeks the law which governs or explains the phenomena, and when this is found, the records of isolated instances of its action usually become of small importance so far as that law is concerned. We care little now for the records of the chemical experiments of a century ago, and the many detailed accounts of the earlier cases of the use of ether or chloroform are of so little interest at the present time that it is not worth while to refer to them in a bibliography of the subject. And although much has been done towards classifying and indexing our medical records (more, in fact, than most physicians suppose), still, as Helmholtz points out, such knowledge as this hardly deserves the name of science, since it neither enables us to see the complete connection nor to predict the result under new conditions yet untried.

Do I seem to depreciate the value of the thoughts which our masters have left us, and which have furnished the foundations on which we build?--or to undervalue the importance of the great medical libraries in which are stored these thoughts?---or to speak slightly of the utility of the catalogues, and indexes, and bibliographies, without which such libraries are trackless and howling wildernesses? If so, I have said what I did not mean to say. The subject has been considered from the point of view of what used to be called the division of labor, but which now I suppose should be called evolution and differentiation; and this has been done because life is short and the art is long---with fair prospect of becoming longer. It is surely unnecessary for me to enter upon any panegyric of books or libraries. As Dr. Holmes says: "It is not necessary to maintain the direct practical utility of all kinds of learning. Our shelves contain many books which only a certain class of medical scholars will be likely to consult. There is a dead medical literature, and there is a live one. The dead is not all ancient, the live is not all modern. There is none, modern or ancient, which, if it has no living value for the student, will not teach him something by its autopsy. But it is with the live literature of his profession that the medical practitioner is first of all concerned."

In medicine, as in social science, we must depend for many facts upon the observation of conditions which occur very rarely, and which cannot be repeated at pleasure. I have already alluded to the importance of Nature's vivisections to the physiologist, and a record of a case written a century ago may be just the link that is needed to correlate the results of his experiments of yesterday with existing theories. The case which at first seems unique and inexplicable both receives and furnishes light when compared with ancient records.

A science of medicine, like other sciences, must depend upon the classification of facts, upon the comparison of cases alike in many respects, but differing somewhat, either in their phenomena or in the environment. The great obstacle to the development of a science of medicine is the difficulty in ascertaining what cases are sufficiently similar to be comparable; which difficulty is in its turn largely due to insufficient and erroneous records of the phenomena observed. This defect in the records is largely due---first, to ignorance on the part of observers; second, to the want of proper means for precisely recording the phenomena; and third, to the confused and faulty condition of our nomenclature and nosological classifications.

Let us consider each of these points briefly. Very, very few are the men who can, by and for themselves, see and describe the things that are before them. Just as it took thousands of years to produce a man who could see, what now any one can see when shown him, that the star Alpha in Capricorn is really two separate stars, so we had to wait long before the man came who could see the difference between measles and scarlatina, and still longer for the one who could distinguish between typhus and typhoid. Said Plato, "He shall be as a god to me, who can rightly divide and define," Men who have this faculty, the *Blick* of the Germans, we cannot produce directly by any system of education; they come we know not when or why, "forming a small band, a mere understanding of whose thoughts and works is a test of our highest powers. A single English dramatist and a single English mathematician have probably equalled in scope and excellence of orignal work in their several fields all the like labors of their countrymen put together."*

But cannot we do something to increase the number of observers by telling them what to observe? It is probable that much may be accomplished in this direction, provided that care be taken to limit the field. Manuals of "what to observe at the bedside and in the post-mortem room" are very well in their way, but can never be made to reach the great majority of the profession, nor would they be of much use if they did. If a few, a very few, distinct specific questions are brought to the attention of the general practitioner, he will often be on the alert for their

* Iles, "Mathematics in Evolution." *Pop. Sci. Monthly*, 1876, ix., page 207.

answer. And it should be remembered that chance may present to the most obscure practitioner an opportunity for observation which the greatest master may never meet.

The great difficulty is to get such questions prepared. They must relate to matters that are just in the nebulous region between the known and unknown---to points not yet clear, but of which we know enough to make it probable that by observing in a definite direction they can be made clear; and to prepare them requires not only knowledge, but a certain reaching out beyond knowledge. It usually happens that the man who has this faculty strives to answer his questions himself, and no doubt he can usually do it better than another. But much can be done towards defining and marking out what we do not know, and this has been a powerful aid to the progress of physiology in recent years.

I have had occasion to refer to this in speaking of Professor Foster's work on physiology, in each section of which an attempt is made to separate that which may be considered as proved from that which is merely probable; and thus almost every page becomes suggestive of work to be done.

Another example of what I mean will be found in a paper on the collection of data at autopsies by Professor H. P. Bowditch, of Boston (*Trans. Mass. Med. Legal Soc.*, i., 1880, page 139). Taking the results of an investigation into the absolute and relative size of organs at different periods of life, and in connection with different morbid tendencies, recently published by Professor Beneke, of Warburg,---Dr. Bowditch urges the securing as large a number as possible of such data, and selects certain of Professor Beneke's results for special inquiry; as, for instance, that "the cancerous diathesis is associated with a large and powerful heart, capacious arteries, but a relatively small pulmonary artery, small lungs, well-developed bones and muscles, and tolerably abundant adipose tissue." It can hardly be doubted that those who read the papers of Professors Bowditch and Beneke will be induced to examine things which before would have had for them no interest, and therefore to make and record observations in pathological anatomy which otherwise would have been lost.

The second difficulty referred to, viz., the want of means for making accurate records, is one that is yearly growing less. It behoves us to be modest in our predictions as to what may be accomplished in the future towards the solution of our Sphynx's riddle. We see as through a glass darkly, and, except through the glass, in nowise; but at least we have made such progress that what we do see we can to a great extent so record that our successors yet unborn can also see: and it is owing to this fact that a part of the medical literature of the last quarter of the nineteenth century will be more valuable than all that has preceded it.

The word-pictures of disease traced by Hippocrates and Sydenham, or even those of Graves and Trousseau, interesting and valuable as they are, are not comparable with the records upon which the skilled clinical teacher of the present day relies. Yet how imperfect in many cases are even the best of these records as compared with what might be given with the resources which we have at our command. The temperature-chart has done away with the errors which necessarily follow attempts to compare the memory of sensations perceived last week with the sensations of to-day; and the balance and the burette enable us to estimate with some approach to precision the tissue-changes of our patients by the records of change in the excretions which they furnish; but we must still trust to our memory, or to the imperfect descriptions of what others remember, when we attempt to compare the results obtained on successive days by auscultation or percussion, although the phonograph and microphone strongly hint to us the possibility of either accurately reproducing the sounds of yesterday, or of translating them into visible signs, perhaps something like the dot and dash record of the telegraph code, which could then be given to the press, and so compared with each other by readers at the antipodes.

We are beginning to count the blood corpuscles, and to use photomicrography, but we do not yet apply the latter process to the former so as to enable every reader to count for himself.

The connections of medicine with the physical sciences are yearly becoming closer, and the methods by which these sciences have been brought to their present condition are those by which progress has been, and is to be, made in therapeutics as well as in diagnosis or in physiological research. These methods turn mainly upon increasing the delicacy and accuracy of measurements: of expressing manifestations of force in terms of another force, or of dimension in space or time. The balance and the galvanometer, the microscope and the pendulum, the camera, the sphygmograph and the thermometer, are some of the means by which investigators, at the bedside and in the laboratory, are seeking to obtain records which shall be independent of their own sensations or personal equations, which shall be taken and used as expressing, not opinions but facts; and with every addition to or improvement in, these means of measurement and record, the the field of observation widens, and new and more reliable materials are furnished for the application of logical and mathematical methods.

Upon the third difficulty which has been referred to, viz., our confused and defective terminology, I need not dwell. "Science," said Condillac, "is a language well made;" and though this is far from being the whole truth, it is an important part of it. In examining medical reports and statistics, it is necessary to bear constantly in mind that to understand many terms you must know what the individual writer means by them. When, for example, we find in such statistics a certain number of deaths attributed to gastroenteritis, or croup, or scrofula, we have to take into account the country, the period, and the individual author, in order to get even a fair presumption as to what is meant.

The three difficulties which have been referred to, although the most important, are by no means the only causes of the confusion and imperfection of our records.

Prominent among the minor troubles of the investigator are defective or misleading titles;---and in behalf of the readers and bibliographers of the future I would appeal to authors, and more especially to editors, to pay more attention than many of them do to the matter of titles and indexes. The men to whom your papers are most important, and who will make the best use of them provided they know of their existence, are for the most part hard workers, busy men, who have a right to demand that their literary table shall be provided with properly prepared materials, and not with shapeless lumps.

The editors of transactions of societies, whether these are sent to journals, or published in separate form, often commit numerous sins of omission in the matter of titles. The rule should be that every article which is worth printing is worth a distinct title, which should be as concise as a telegram, and be printed in a special type. If the author does not furnish such a title, it is the editor's business to make it, and he should not be satisfied with such headings as "Clinical Cases," "Difficult Labor," "A Remarkable Tumor," "Case of Wound, with Remarks." The four rules for the preparation of an article for a journal will then be:—1. have something to say; 2. Say it; 3. Stop as soon as you have said it; 4. Give the paper a proper title.

Some societies and editors do not seem to appreciate fully their responsibility for the articles which they accept for publication—a responsibility which cannot be altogether avoided by any formal declaration disclaiming it. This is due to the fact that while the merits of a paper can usually be determined by examination, this is by no means always the case. In every country there are writers and speakers whose statements are received with very great distrust by those best acquainted with them. Supposing these statements to be true, the papers would be of much interest and importance; but the editor should remember that a certain number of readers, and especially those in foreign countries, have no clue to the character of the author, beyond the fact that they find his works in good company. In medical literature, as in other departments, we find books and papers from men who are either constitutionally incapable of telling the simple literal truth as to their observations and experiments, although they may not write with fixed intention to deceive, or from men who seek to advertise themselves by deliberate falsehoods as to the result of their practice. Such men are usually appreciated at their true value in their immediate neighborhood, and find it necessary to send their communications to distant journals and societies in order to secure publication.

I presume that you are all familiar with the peculiar feeling or distrust which is roused by too complete an explanation. The report of a case in which every symptom observed, and the effect of every remedy given, is fully accounted for, and in which no residual unexplained phenomena appear, is usually suspicious, for it implies either superficial observation, or suppression or distortion of some of the facts. A diagramatic representation is usually much plainer than a good photograph, but also of much less value as a basis for farther work.

No fact is more familiar to this audience than the vast extent of the field of the science of to-day---so vast that few may hope to master more than a small part of it, and yet so closely connected that even the small part cannot be fully grasped without some acquaintance with a much wider field.

But little over a hundred years ago, Haller in Gottingen was professor of anatomy, botany, physiology, surgery and obstetrics, and lecturer on medical jurisprudence. At the same time he was writing one review a week, and summing up existing medical science in his Bibliothecæ. To-day any one of these branches requires all the time of the most energetic and learned of our contemporaries, but on the other hand the well-educated medical graduate of to-day could give Haller valuable instructions in each of the branches of which he was professor. It is also true, as I have pointed out, that our actual progress is by no means in proportion to the work done, nor as great as these merely quantitative statements would seem to make it.

Science has been termed "the topography of ignorance." "From a few elevated points we triangulate vast spaces, enclosing infinite unknown details. We cast the lead, and draw up a little sand from abysses we shall never reach with our dredges. If it is true that we understand ourselves but imperfectly in health, it is more signally manifest in disease, where natural actions imperfectly understood, disturbed in an obscure way by half-seen causes, are creeping and winding along in the dark toward their destined issue, sometimes using our remedies as safe stepping-stones, occasionally, it may be, stumbling over them as obstacles." *

* Border Lines of Knowledge," etc, by O. W. Holmes, Boston, 1862, pages 7, 8.

In days of old, when the profession of medicine, or of a single medical specialty, was an inheritance in certain families, a large part of their knowledge, and the efficiency of their remedies was thought to depend upon these being kept a profound mystery. Among the precepts of magic there was no more significant one than that which declared that the communication of the formula destroyed its power, and that hence attempts to reveal the secret must always fail. We have changed all that. Every physician hastens to publish his discoveries and special knowledge, and a good many do the same by that which is not special, or which is not knowledge. For the individual, in a degree---for the nation or the race in a much greater degree---the literature produced is the most enduring memorial. The whole result of civilization has been cynically defined as being roughly, three hundred million Chinese, two hundred million natives of India, two hundred million Europeans and North Americans, and a miscellaneous hundred million or two of Central Asians, Malays, South Sea Islanders, etc., and over and above all the rest the Library of the British Museum. This is the net result of an indefinitely long struggle between the forces of men and the weights of various kinds in the attempt to move which these forces display themselves."*

And thus in our great medical libraries each of the folios or quaint little black-letter pamphlets which mark the first two centuries of printing, or of the cheap and dirty volumes of more modern days, with their scrofulous paper and abominable typography, represents to a great extent the life of one of our profession and the fruit of his labors, and it is by the fruit that we know him.

After stating that modern physicists have concluded that the sun is going out, that the earth is falling into the sun, and therefore that it and all things in it will be either fried or frozen, Professor Clifford concludes that "our interest lies so much with the past as may serve to guide our actions in the present, and with so much of the future as we may hope will be affected by our

* Liberty, Equality, and Fraternity," by James Fitz James Stephen, New York, 1873, page 178.

actions now. Beyond that we do not know and ought not to care. Does this seem to say, Let us eat and drink, for to-morrow we die? Not so, but rather, Let us take hands and help, for this day we are alive together." To this I join a verse from the Talmud which will remind you of the first aphorism of Hippocrates, and is none the worse for that: "The day is short and work is great,—the reward is also great, and the master presses. It is not incumbent on thee to complete the work, but thou must not therefore cease from it."

